# Symptoms of Major Depressive Disorder and Their Impact on Psychosocial Functioning in the Different Phases of the Disease: Do the Perspectives of Patients and Healthcare Providers Differ?

**DOI:** 10.3389/fpsyt.2020.00280

**Published:** 2020-04-24

**Authors:** Michael Cronquist Christensen, Chiew Meng Johnny Wong, Bernhard T. Baune

**Affiliations:** ^1^ Medical Affairs, H. Lundbeck A/S, Valby, Denmark; ^2^ Biostatistics, Lundbeck Singapore Pte. Ltd., Singapore, Singapore; ^3^ Department of Psychiatry and Psychotherapy, University of Münster, Münster, Germany; ^4^ Department of Psychiatry, Melbourne Medical School, University of Melbourne, Melbourne, VIC, Australia; ^5^ The Florey Institute of Neuroscience and Mental Health, University of Melbourne, Melbourne, VIC, Australia

**Keywords:** major depressive disorder, psychosocial functioning, cognitive symptoms, treatment phase, acute, post-acute, remission, recovery

## Abstract

This analysis was undertaken to examine the relationship between different symptoms of major depressive disorder (MDD) and psychosocial functioning from the perspectives of patients and healthcare providers (HCPs) across the different phases of the disease (acute, post-acute, and remission). Data regarding symptoms of MDD and psychosocial functioning, assessed by an adapted version of the Functioning Assessment Short Test (FAST) scale, were elicited *via* an online survey from 2,008 patients diagnosed with MDD (based on their personal experience of the disease) and 3,138 patients observed by 1,046 HCPs (based on individual patient records). Correlations between patient-reported and HCP-reported MDD symptoms and impairment of psychosocial functioning were assessed by multivariate regression analysis. The population comprised 1,946 patient respondents and 3,042 HCP-reported patients. Patients reported experiencing a wider range of symptoms and greater impairment of functioning than reported by HCPs across all phases of the disease. At the domain level, only cognitive symptoms were found to be significantly associated with functioning during the acute phase from the perspective of patients, while from the HCPs’ perspective both mood and cognitive symptoms significantly impacted functioning in this phase. Significant associations were seen between mood, physical, and cognitive symptom domains and functioning in both cohorts during the post-acute and remission phases. Differences in associations between *individual* MDD symptoms and functioning were also observed between the two cohorts across all disease phases; in particular, HCPs found that more physical symptoms impacted functioning during remission than did patients. In summary, the results suggest that perceptions of MDD symptoms and the associations between these symptoms and functioning differ significantly between patients and HCPs across all phases of the disease. These findings further highlight the need for improved communication between patients and HCPs in order to set appropriate treatment goals and promote symptomatic and functional recovery in MDD.

## Introduction

Major depressive disorder (MDD) is a complex and multidimensional condition (1), which is associated with significant impairment of psychosocial functioning and health-related quality of life (2–4). In addition to depressed mood and/or anhedonia, patients with MDD may experience a wide range of physical and cognitive symptoms (5). Clinical presentation is highly heterogeneous, and large variations in symptom profiles exist between individual patients (6, 7).

Both symptomatic and functional recovery are required if patients with MDD are to return to productive and fulfilling daily lives (8–10); however, achieving these treatment goals remains challenging in clinical practice. Approximately 50% of patients with MDD do not respond adequately to initial antidepressant treatment (11), with patients who achieve only partial response experiencing significant impairments in overall functioning compared with those who achieve remission (12). Residual symptoms during periods of remission have been shown to be strong predictors of subsequent relapse in patients with MDD (13–18).

Successful management of MDD necessitates shared decision-making between patients and healthcare providers (HCPs) to set appropriate treatment goals (19). However, available data suggest that patients and HCPs differ in their views as to what they consider important for recovery from MDD (20–23). We have previously reported results of a large, international, online survey undertaken to assess potential differences in perceptions of MDD symptoms and treatment priorities between patients and HCPs across the different phases of the disease (23). We found that patients more frequently reported mood, physical, and cognitive symptoms than HCPs reported with regard to their patients, particularly during the post-acute and remission phases of MDD. Patients also reported greater impact of symptoms on psychosocial functioning than did HCPs. In addition, patients more frequently reported inadequately treated symptoms across all domains and phases of MDD compared with HCPs. While alleviation of mood symptoms was found to be a priority for both patients and HCPs in the acute phase of MDD, patients also reported the need for improvements in physical and cognitive symptoms in order to address the impact of MDD on psychosocial functioning. In contrast, HCPs underestimated across all disease phases the number of patients who wanted physical and cognitive symptoms to be addressed.

This observed discordance between patient and HCP perceptions has important implications for the diagnosis and management of MDD. Greater alignment of patient and HCP perceptions of depression impact and treatment goals may be expected to facilitate functional recovery and improve long-term outcomes. This analysis of the survey data was undertaken to more comprehensively explore the relationship between individual symptoms of MDD and psychosocial functioning from the perspectives of both patients and HCPs across the different phases of the disease using multivariate regression analysis.

## Methods

### Study Design

This is a detailed analysis of data from an online survey undertaken between February 14 and March 28, 2017, in patients with MDD and HCPs (primary care physicians, psychiatrists, and neurologists) treating patients with MDD in eight countries (Brazil, Canada, Mexico, South Korea, USA, France, Italy, and Spain). Study design, inclusion/exclusion criteria, and survey development have been reported in detail previously (23). In brief, respondents were recruited through existing online panels of consumers and HCPs. Participating patients were required to be ≥18 years old (≥25 years in the USA) and have a profile indicative of a history of depression during the past 12 months. Patients confirmed that they had been diagnosed with depression by a physician and were either currently using prescribed antidepressant medication for their depression or had used medication to treat depression in the past 3 months. Patients were asked to carefully review three statements describing different phases of depression and indicate which of these best described their current disease state.

Participating HCPs were required to undergo a rigorous screening and authentication process that included an introductory telephone call and validation of identity. Participating HCPs were also required to have treated and managed a minimum number of MDD patients per month: primary care physicians, ≥15 patients (≥10 in Brazil); psychiatrists, ≥40 patients; and neurologists, ≥25 patients. Other HCP eligibility criteria included: ≥75% of working hours to be spent in direct patient care; prescribing antidepressants to ≥75% of their patients with MDD; and, in the USA, <10% of patients living in long-term care facilities. For inclusion, HCPs were also required to confirm that they were able to refer to case records for individual patients matching each of the three different disease phases during completion of the survey.

Patients and HCPs were excluded from survey participation if they were employed by or affiliated with a pharmaceutical company, marketing agency, or any other unsuitable agency (*e.g.*, a government agency, health insurance company, or pharmacy/drug store).

All respondents had previously consented to participate in research. Consent was also obtained from all respondents specifically for this survey before participation. Respondents were offered reward points for participating in the survey, which could be redeemed for PayPal credit, gift vouchers, or air miles, or donated to charity.

### Survey Assessments

The full questionnaires have been published previously (23). The 25-minute online patient survey elicited responses based mainly on current experiences with MDD, including disease phase (*i.e.*, acute, post-acute, and remission), type of physician consulted, type and relative importance of symptoms experienced, functional impairment, and antidepressant treatment received. The 30-minute online HCP survey required completion of three patient record forms corresponding to the last patient treated for each phase of depression; HCPs were requested to refer to patient records when completing the patient record forms.

Respondents were asked to indicate all the symptoms experienced in the current disease phase, from a list of 23 symptoms. The total symptom score comprised the number of symptoms experienced (*i.e.*, 0–23). The score for each domain was also calculated, based on the number of symptoms experienced within the domain (mood 0–7, physical 0–10, and cognitive 0–6).

Both the patient and HCP surveys incorporated the Functioning Assessment Short Test (FAST) questionnaire, the wording of which had been slightly amended from the original version to facilitate data collection. The FAST questionnaire is a brief instrument designed to assess the main problems in daily functioning experienced by psychiatric patients (24), including those with MDD (25, 26). It comprises 24 items that assess impairment or disability across six domains of psychosocial functioning: autonomy, occupational functioning, cognitive functioning, financial issues, interpersonal relationships, and leisure time. Respondents were asked to select the degree of difficulty (‘no difficulty,’ ‘mild difficulty,’ ‘moderate difficulty,’ ‘severe difficulty,’ or ‘don’t know’) associated with each item. Total FAST score ranges from 0 to 72; higher scores indicate greater disability.

### Statistical Analysis

For patients and HCPs, the population for analysis comprised all respondents who met all inclusion criteria and no exclusion criteria and completed their respective online survey. Respondents were excluded from the final sample for analysis if they discontinued the survey for any reason, were deemed to have provided identical answers to a series of questions, completed the survey in less than 40% of the mean time taken by other respondents, or provided nonsensical and/or open-ended responses. Respondents who selected ‘don’t know’ for five or more items on the FAST questionnaire were removed from the analysis of the FAST total scores, but their other questionnaire responses were included in other descriptive analyses (*e.g.*, analysis of total symptom scores).

Data were analyzed separately for patients and HCPs. Summary statistics (mean and standard deviation for continuous variables, and counts and percentages for categorical variables) were estimated for demographic and clinical characteristics, symptoms experienced, and the FAST scores. Multivariate regression analysis was applied to examine the significance of correlations between MDD symptom scores (at the domain and individual level) and the FAST total score. Data on age, sex, country of origin, and level of education were included as covariates. All statistical tests were two-sided; the significance level was 5%. Analyses were performed using the statistical software R (version 3.5.0) (27).

## Results

### Survey Population

Of the 14,048 patients identified as having depression through initial screening and invited to participate, 2,379 took part in the survey (16.9%). Of the 2,428 HCPs invited to participate, 1,223 took part in the survey (50.4%). A total of 2,008 patients and 1,046 HCPs (366 primary care physicians, 650 psychiatrists, and 30 neurologists) accurately completed the surveys, with HCPs providing data for a total of 3,138 patients. The population for the multivariate analysis comprised 1,946 patient respondents and 3,042 HCP-reported patients; 62 and 96 patients having been excluded from the two cohorts, respectively, as they had missing FAST total score. In terms of disease phase, 406, 767, and 773 responses were included for the patient-reported cohort and 1,005, 1,017 and 1,020 for the HCP-reported cohort for the acute, post-acute, and remission phases, respectively.

### Baseline Characteristics

Sociodemographic characteristics at baseline were similar in the two patient cohorts (**Table 1**). In both cohorts, the majority of patients were female and ≥31 years old. No differences were seen between the two cohorts in terms of education and working status. Most patients were currently receiving antidepressants for MDD (80% in the patient-reported cohort and 96% in HCP-reported cohort).

**Table 1:** Baseline sociodemographic characteristics and symptom profile.

	Patient-reported cohort (*N* = 2,008)	HCP-reported cohort (*N* = 3,138)
**Sex**, *n* (%)		
Male	793 (39.5)	1,133 (36.1)
Female	1,215 (60.5)	2,005 (63.9)
**Mean age**, years (SD)	45.2 (13.3)	44.3 (13.6)
18–30 years, *n* (%)	341 (17.0)	560 (17.8)
31–50 years, *n* (%)	900 (44.8)	1,564 (49.8)
≥51 years, *n* (%)	767 (38.2)	1,014 (32.3)
**Education status**, *n* (%)		
No qualifications/other/don’t know	61 (3.0)	283 (9.0)
Vocational qualification	349 (17.4)	397 (12.7)
High school graduate	608 (30.3)	1,044 (33.3)
Undergraduate	683 (34.0)	982 (31.3)
Postgraduate	307 (15.3)	432 (13.8)
**Working status**, *n* (%)		
Student/homemaker/retired/unemployed	829 (41.3)	1,277 (40.7)
Part-time	272 (13.5)	464 (14.8)
Full-time	907 (45.2)	1,397 (44.5)
**FAST total score**, mean (SD)^a^		
Overall	40.5 (16.2)	35.4 (17.2)
Acute phase	48.7 (13.7)	47.8 (12.3)
Post-acute phase	43.4 (14.2)	35.9 (13.8)
Remission phase	33.4 (16.2)	22.7 (15.4)
**Symptom score**, mean (SD)		
Total	9.0 (5.3)	7.0 (5.2)
Mood	3.3 (2.3)	2.4 (2.1)
Physical	3.8 (2.2)	3.0 (2.3)
Cognitive	2.0 (1.8)	1.6 (1.6)

Percentage data refer to the proportion of patients.

a Data available for all patients/HCP patient case records, excluding those who stated ‘don’t know’ to ≥5 statements. For patient-reported cohort, N = 1,946 (acute, n = 406; post-acute, n = 767; remission, n = 773). For HCP-reported cohort, N = 3,042 (acute, n = 1,005; post-acute, n = 1,017; remission, n = 1,020).

FAST, Functioning Assessment Short Test; HCP, healthcare professional; SD, standard deviation.

**Figure 1:**
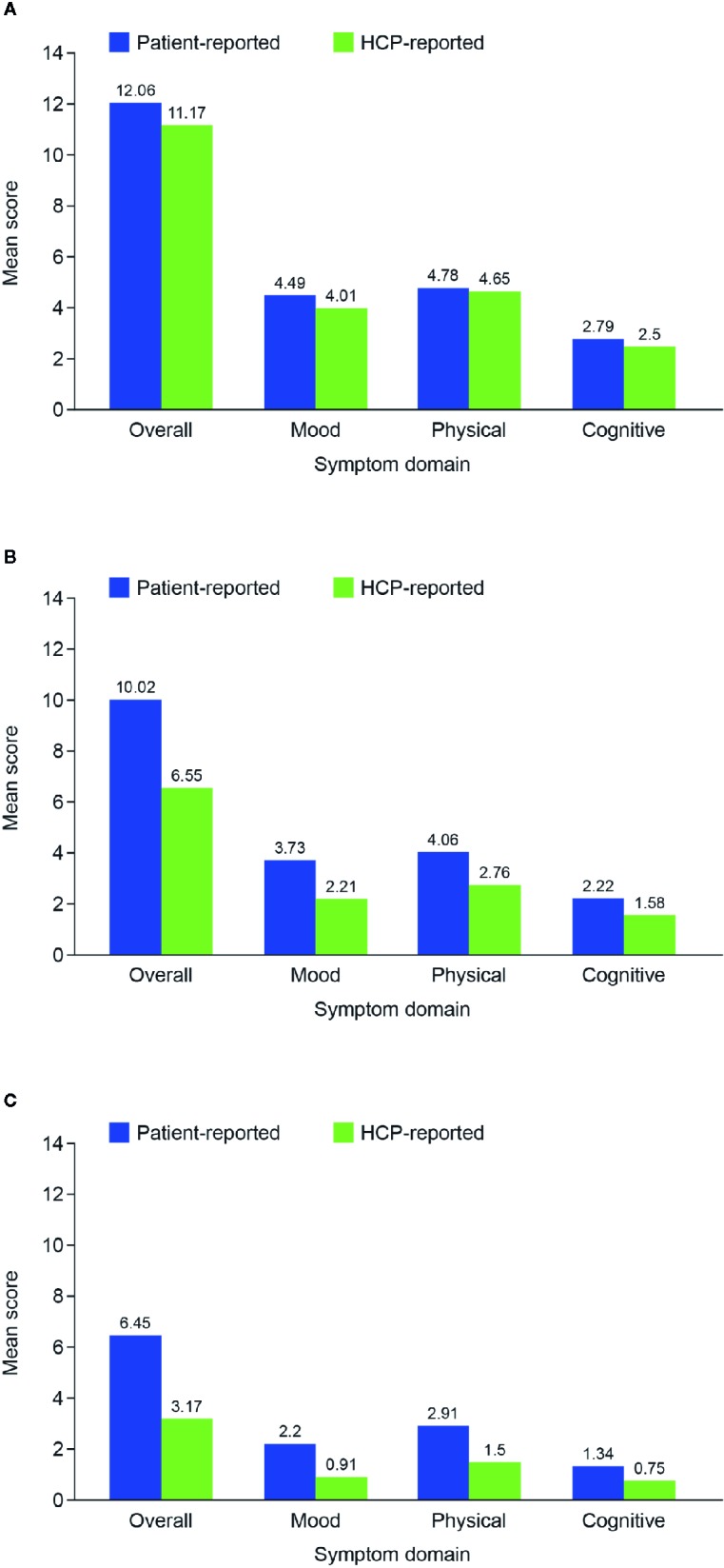
Mean overall and symptom domain scores in the two patient cohorts according to disease phase: **(A)** acute; **(B)** post-acute; and **(C)** remission. HCP, healthcare provider.

**Figure 2:**
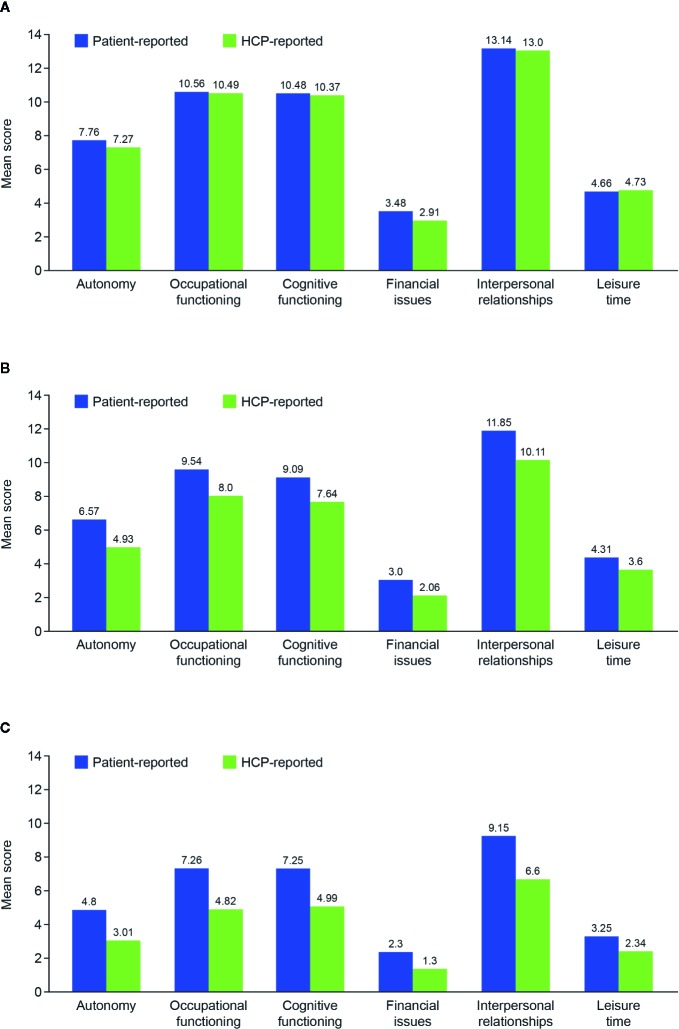
Mean Functioning Assessment Short Test score for each domain in the two patient cohorts according to disease phase: **(A)** acute; **(B)** post-acute; and **(C)** remission. HCP, healthcare provider.

Mean total symptom score and all symptom domain scores were higher in the patient-reported cohort than in the HCP-reported cohort (**Table 1**). This trend was apparent across all disease phases (**Figure 1**). Overall mean total FAST score was also higher in the patient-reported cohort than in the HCP-reported cohort (**Table 1**). Mean total FAST scores were generally similar in both cohorts for the acute phase but higher in the patient-reported cohort than in the HCP-reported cohort for the post-acute and remission phases. Mean scores for individual FAST domains were also similar in the two cohorts for the acute phase, but were generally higher across all domains in the patient-reported cohort compared with the HCP-reported cohort for the post-acute and remission phases (**Figure 2**).

### Multivariate Analysis

Multivariate analysis of associations between MDD symptom domains (physical, mood, and cognitive) and total FAST score showed only cognitive symptoms to be significantly associated with functioning in the patient-reported cohort in the acute phase of MDD. However, both mood and cognitive symptoms were found to be significantly associated with patient functioning in this disease phase in the HCP-reported cohort (**Table 2**). Significant associations were seen between all three symptom domains and functioning in both cohorts during the post-acute and remission phases.

**Table 2:** Associations between FAST total score and symptoms reported by phase of depression (acute, post-acute, and remission) in the patient- and HCP-reported cohorts; regression coefficients at the symptom domain level.

	Patient-reported cohort			HCP-reported cohort		
	Acute (*n* = 406)	Post-acute (*n* = 767)	Remission (*n* = 773)	Acute (*n* = 1,005)	Post-acute (*n* = 1,017)	Remission (*n* = 1,020)
**Symptom domain**						
Mood	0.52	1.02*	1.40**	0.52*	1.00*	1.64**
Physical	0.32	0.89*	1.09*	0.36	0.99*	2.10**
Cognitive	1.72**	1.96**	2.16**	1.19**	1.44**	2.74**
**Age**	−0.08	−0.17**	−0.20**	−0.03	−0.05	−0.06
**Sex** (Ref: Male)	−2.20	−0.62	0.37	1.92*	0.23	2.39*
**Education** (Ref: No qualifications/other/don’t know)						
Vocational qualification	1.14	0.56	0.46	0.43	−2.98	0.61
High school graduate	1.96	0.23	−0.65	0.57	−4.61*	−1.96
Undergraduate	0.95	0.90	−1.00	−0.65	−4.32*	−4.53*
Postgraduate	2.70	0.01	−1.51	−1.79	−5.21*	−5.03*
**Country** (Ref: Brazil)						
Canada	−5.14*	−5.60*	−7.43*	−1.47	−4.57*	−8.12**
Spain	−0.97	−2.39	−2.39	−0.03	−0.60	−3.83*
France	−2.98	−0.96	−3.46	2.95	2.45	−2.52
Italy	−3.39	−1.23	−0.70	1.05	0.81	−1.42
Mexico	−4.47	−1.59	−0.44	1.06	−0.93	−3.37
South Korea	2.82	1.93	4.76*	8.45**	2.06	−2.54
USA	−8.96*	−7.53*	−8.38**	1.37	−2.58	−5.55*

*p < 0.05; **p < 0.0001.

Number of patients in the regression model is not identical to the total number of patients included in each cohort due to missing data. FAST, Functioning Assessment Short Test; HCP, healthcare professional.

Differences in associations between individual symptoms of MDD and functioning were also observed between the two cohorts across all disease phases (**Table 3**). In terms of mood symptoms, significant associations were seen in the patient-reported cohort for ‘lack of interest in general’ in the acute phase and ‘low self-esteem’ in the remission phase. In the HCP-reported cohort, significant associations were seen for ‘feelings of guilt or worthlessness’ and ‘suicidal thoughts’ in the acute phase, for ‘low self-esteem’ in the post-acute phase, and for ‘lack of confidence’ in the remission phase. No associations were seen between any individual physical symptoms and functioning in either cohort in the acute phase; however, significant associations between physical symptoms and functioning were seen in both cohorts during the post-acute and remission phases. Of note, significant associations between physical symptoms and functioning were more frequent in the HCP-reported cohort than in the patient-reported cohort during the remission phase. Significant associations were seen between at least one cognitive symptom and functioning in both cohorts during all disease phases. For the patient-reported cohort, significant associations were seen for the symptoms of ‘difficulty in making plans’ during the acute phase, ‘slowness of thinking’ and ‘difficulty concentrating’ during the post-acute phase, and ‘difficulty concentrating’ and ‘difficulty in making plans’ during the remission phase. For the HCP-reported cohort, significant associations were seen for ‘forgetfulness/difficulty remembering’ during the acute and post-acute phases, and for ‘slowness of thinking’ and ‘difficulty in making plans’ during the remission phase. The magnitude of effect of the individual symptoms on functioning did not differ significantly in either cohort.

**Table 3:** Associations between FAST total score and symptoms reported by phase of depression (acute, post-acute, and remission) in the patient- and HCP-reported cohorts; regression coefficients at the individual symptom level.^a^

	Patient-reported cohort			HCP-reported cohort		
	Acute (*n* = 406)	Post-acute (*n* = 767)	Remission (*n* = 773)	Acute (*n* = 1,005)	Post-acute (*n* = 1,017)	Remission (*n* = 1,020)
**Mood symptoms**						
Feeling sad/low	−3.20	−0.21	0.56	−0.97	0.55	−0.46
Lack of interest in general	3.94*	1.64	1.80	0.41	1.34	1.67
Unable to take pleasure in things	−0.49	0.91	0.24	−1.21	1.73	1.18
Low self-esteem	−0.05	2.04	3.20*	0.16	2.44*	1.44
Lacking in confidence	1.82	1.04	1.34	0.06	0.50	3.12*
Feelings of guilt or worthlessness	−0.69	0.08	1.33	2.01*	−0.74	3.16
Suicidal thoughts	1.79	1.93	−1.31	3.02*	2.34	−0.62
**Physical symptoms**						
Lack of energy	1.99	0.60	2.68*	1.06	1.76*	4.01*
Reduced activity	2.40	3.45*	−0.24	1.49	0.99	3.52*
Difficulty sleeping	−2.30	2.25*	1.42	0.07	−0.06	−0.74
Feeling tired	−3.03	−0.79	1.29	−0.83	−0.22	3.10*
Waking early in the morning	0.50	−1.13	−0.65	−0.08	0.73	4.78*
Feeling agitated/restless	−0.11	−0.01	1.05	0.39	−0.11	2.60
Weight loss	0.66	2.34	0.28	0.93	2.63	−2.04
Loss of sex drive	1.74	1.06	2.00	1.07	1.83*	3.12*
Loss of appetite	1.16	0.36	3.06	0.0002	2.37	3.54
Depression worst in the morning	0.59	1.12	−0.83	0.54	1.51	−3.09
**Cognitive symptoms**						
Forgetfulness/difficulty remembering	−0.68	0.97	1.26	2.38*	2.98*	2.58
Slowness of thinking	1.60	3.10*	0.86	1.57	1.24	5.31*
Slowness in physical movements	2.55	2.14	−0.98	0.75	2.28	2.08
Difficulty concentrating	2.83	3.01*	4.62**	0.91	1.39	1.15
Difficulty to prioritize/make decisions	−0.58	0.81	1.61	0.66	−0.38	2.15
Difficulty in making plans	4.47*	1.06	3.85*	0.59	1.36	3.12*

*p < 0.05; **p < 0.0001.

a Age, sex, country of origin, and level of education were included as covariates in the multivariate regression analysis (data not shown).

Number of patients in the regression model is not identical to the total number of patients included in each cohort due to missing data. FAST, Functioning Assessment Short Test; HCP, healthcare professional.

## Discussion

This multivariate analysis was undertaken to explore statistically how perceived individual symptoms of MDD and psychosocial functioning differ between patients and HCPs across the different phases of the disease. We found that patients reported more symptoms of MDD and greater impairment of functioning than HCPs across all disease phases. Our findings also highlight the importance of cognitive symptoms in patients with MDD across all disease phases. Indeed, in patients with MDD, at the domain level only cognitive symptoms were found to be significantly associated with functioning during the acute phase of MDD; however, significant associations between both mood and cognitive symptoms and functioning were observed in the HCP-reported cohort during this disease phase. Differences in associations between *individual* MDD symptoms and functioning were also observed between the two cohorts across all disease phases, with more cognitive symptoms found to be associated with functioning in the patient-reported cohort and more physical symptoms of MDD associated with functioning in the HCP-reported cohort.

Our results support earlier findings reporting important differences in perspectives between patients and HCPs regarding what “success looks like” for the treatment of depression. Demyttenaere et al. (22) found patients’ top three priorities to be to what extent life is meaningful, level of enjoyment in life, and satisfaction in life, while HCPs ranked addressing negative feelings (blue mood, despair, anxiety, depression), feeling down/depressed/hopeless, and little interest or pleasure in doing things as their top three priorities. These top-ranking treatment priorities in patients were similar to those reported by Zimmerman et al. (28), who found the presence of positive mental health, feeling your usual self, and restoring functioning to be the most important priorities from the patients’ perspective. Alleviation of depressive symptoms was ranked only sixth (28). Our finding of cognitive symptoms being the only significant symptom domain significantly associated with functioning in the acute phase of MDD from a patient perspective, unlike both cognitive and mood symptoms from an HCP perspective, is intriguing in this regard.

Cognitive symptoms are prevalent during the course of MDD, and important for patient functioning. In one prospective study, cognitive symptoms were found to be present for 85–94% of the duration of depressive episodes and 39–44% of the duration of periods of remission (29). The presence of cognitive symptoms affects patient functioning broadly, including difficulty in maintaining performance at work, experiencing household and financial strains, and difficulty in participating in social life (30–32). Improvements in these symptoms have been shown to precede improvements in functional outcome, even after adjustment for depressive symptom severity (33–35). Our findings may indicate that patients experience the functional consequences of cognitive symptoms in the short term to a much greater extent than realized or observed by HCPs and therefore report them as significant over and above depressive symptoms.

Patient-centered consulting approaches allow treatment to be based on individual patient symptoms and preferences, which may result in improved outcomes (36, 37). Other studies have also reported low recognition by HCPs of cognitive symptoms in patients with MDD (38–40). A survey of Italian psychiatrists showed that, although psychiatrists considered cognitive symptoms among the most relevant residual symptoms in MDD, these symptoms were not always taken into consideration when selecting antidepressant therapy (38). This is of clinical significance as currently available antidepressants have been shown to have differential effects in terms of improving cognitive symptoms in patients with MDD (41).

The presented research has strengths and limitations, which have been described in detail previously (23). In brief, this is the first study to our knowledge to investigate real-world differences in perceptions of MDD symptoms between patients and HCPs across the different phases of the disease (acute, post-acute, and remission phases) and their associations with perceived functioning. Furthermore, data were provided by primary care physicians, psychiatrists and neurologists, representing the full spectrum of HCPs likely to be consulted by patients with MDD in real-world practice. Study limitations include the potential for selection and/or response bias, lack of information about comorbid psychiatric disorders and the presence of subthreshold symptoms, and the potential for cultural differences within the survey population. In terms of this analysis, it should again be noted that comparisons between the two patient cohorts should be interpreted with caution as patient- and HCP-provided responses were unmatched (*i.e.*, patients who participated in this survey were not the same patients as those described by the HCPs). In addition, as the survey by definition is cross-sectional, the data presented here cannot be used to draw conclusions about any causal relationship between symptoms of MDD and psychosocial functioning.

## Conclusions

In summary, the results suggest that patients and HCPs differ in their perceptions of MDD symptoms and their impact on functioning across all phases of the disease. In particular, patients emphasized cognitive rather than mood and physical symptoms in the acute phase of the disease, while HCPs were more likely than patients to associate more specific physical symptoms with functioning during the remission phase. These findings further highlight the need for improved communication between patients and HCPs in order to set appropriate treatment goals based on the individual’s specific symptom profile and promote symptomatic and functional recovery in patients with MDD.

## Data Availability Statement

The datasets presented in this article are not readily available given the informed consent provided by survey participants. Requests to access the datasets should be directed to MC.

## Ethics Statement

All respondents (patients and HCPs) accepted the online panel partners’ privacy policies and terms and conditions when they signed up to become a member of the panel. They thus provided consent to receive invitations to participate in market research, and their consent was sought again to participate in this particular study. The market research protocol was not formally approved by a medical ethics committee.

## Author Contributions

MC and BB were both instrumental in the development of the study, study design, analysis plan, and interpretation of data. CW undertook the statistical analysis and contributed to data interpretation. All authors were involved at all stages of manuscript development and approved the final version.

## Funding

This study was funded by H. Lundbeck A/S, who contributed to the data analysis, review of the data, and review of the manuscript.

## Conflict of Interest

This study was funded by H. Lundbeck A/S, who contributed to the data analysis, review of the data, and review of the manuscript.

MC is an employee of H. Lundbeck A/S. CW is an employee of Lundbeck Singapore Pte. Ltd. BB has received speaker/consultation fees from AstraZeneca, Lundbeck, Pfizer, Takeda, Servier, Bristol-Myers Squibb, Otsuka, and Janssen-Cilag.
